# Family Medicine Practice as Learning Environment: A Medical Student Evaluation in Switzerland

**DOI:** 10.2147/AMEP.S492834

**Published:** 2024-12-21

**Authors:** Stefania Di Gangi, Oliver Senn, Andreas Plate

**Affiliations:** 1 Institute of Primary Care, University of Zurich and University Hospital of Zurich, Zurich, Switzerland

**Keywords:** family medicine practice, undergraduate medical education, teaching placement, student perception, quality evaluation

## Abstract

**Introduction:**

Improving the quality of teaching placements in family medicine practice (FMP) could help to address the shortage of primary care physicians. This study aims to investigate students’ evaluations of first-exposure FMP placements, to identify clusters of FMPs that might need to improve their placement quality, and to analyze students’ perceptions of the FMP as a learning environment.

**Methods:**

The design was a cross-sectional survey study, including all fourth-year undergraduate medical students at the University of Zurich, Switzerland, who completed a mandatory placement in FMP during 2019–2022. The placements consisted of individual teaching and training in the same FMP for 8 half days within an academic year. The primary outcome was the student Likert scale rating of the 14 key questions as indicators of teaching placement quality. Based on these indicators, cluster analysis was used to identify groups of FMPs with the potential for quality improvement. A framework analysis was used to analyze the students’ perceptions.

**Results:**

A total of 713 students (response rate 81%) and 249 FMPs (median [interquartile range]: 2 [1,4] students per FMP) were included. Overall, 86% of the students were satisfied with the placement, and 95% reported that the placement gave them realistic insight into FMP work. A cluster of 25 (10%) FMPs that may improve placement quality was identified. Students most liked the opportunity to gain FMP skills, insight into FMP work, and establishment of patient relationships.

**Conclusion:**

Our study described students’ experiences with FMP and a method for teaching evaluation to identify FMPs that may benefit from interventions to improve their learning environment. This could upgrade the medical education offered and increase the interest in family medicine as a response to the shortage of primary care physicians.

## Introduction

Placements of medical students in family medicine practice (FMP), including general practitioners, internal medicine, and pediatric practices, are common in undergraduate medical training^1^ and there is evidence^2^ that they increase students’ interest in the career. Students receive one-on-one teaching and feedback from their tutors and are not only introduced to the FMP setting but also learn how to deal with different patients, chronically ill or multi-morbid,^3–5^ and acquire clinical skills as well, if not better, as in hospitals.^6^ The major benefits of the FMP learning environment include learners’ participation in diagnosis and management planning, developing a patient-centred approach to care, and shared decision-making.^5^,^7^ Undergraduate primary care exposure should challenge students, testing not only their communication skills but also their clinical reasoning, diagnostic, ethical, and management competences.^8^

Being the first undergraduate students’ exposure to the practical aspects of family medicine, these placements could influence later decisions to undertake further placements or training in FMP, and thus determine future career choices in FMP.^9^ Primary care workforces are declining globally,^10^,^11^ in particular in Switzerland, where one in four physicians is aged 60 or over and expected to retire within a few years.^12^ In addition, FMP plays a crucial role in the health-care system, but medical students do not consider FMP as a career of high interest and prestige.^13–17^ Therefore, improving the supervising and teaching of medical students in FMP placements is not only a matter of education, but also of public policy. In fact, primary healthcare should be strengthened as recommended by the World Health Organization (WHO).^18^

Medical students’ learning experiences should be understood, and student satisfaction should be evaluated. This could help universities and medical educators to improve undergraduate medical education and adapt it to the current needs of society and the healthcare system. This could increase students’ interest in FMP careers and address the shortage of primary care physicians. In addition, being able to identify which FMPs could benefit from programs to improve the quality of placements could also make improvements more efficient. Previous research has investigated students’ perceptions of the quality of primary care placements,^3^,^4^,^6^,^7^,^19–25^ but little is known about Switzerland. With this study, we expect to gain further insight into how to improve placement quality, taking into account that students are spread among many practices and FMP settings are not standardized. This study aimed to investigate how students experienced placements as learning environments for acquiring and practicing FMP skills, and to identify FMPs that could benefit from programs to improve placement quality, using a multidimensional approach that takes into account the number of students per teaching practice. In addition, we investigated how placement characteristics are associated with student satisfaction.

## Materials and Methods

### Setting

In Switzerland, undergraduate medical education, as generally defined,^26^ lasts six years, including both bachelor and master programs of three years each. Placements in FMP are part of a special program to increase the capacity of medical education and improve interprofessional collaboration and primary care.^27^ In this study we focused on the first-exposure FMP placement at the University of Zurich, mandatory for fourth-year undergraduate medical students, which consists of eight visits of half a working day (four hours) within an academic year in the same FMP. During this mandatory placement, students first observe the FMP at work and ask questions. Students are then given the opportunity to take medical histories, examine patients, perform diagnostics (eg, electrocardiogram (ECG), X-ray), and discuss therapies. This placement also provides the opportunity to practice blood draws or injections, as in Switzerland these tasks are usually performed by nurses working in FMP, or to gain experience with medical triage of urgencies. The Institute of Primary Care at the University of Zurich manages the registration process and selects FMPs on the basis of requirements (training, previous teaching experience or willingness to teach, participation in basic didactic courses, capacity). The selected FMPs receive remuneration for the placement of medical students. Each FMP, according to its capacity and resources, can follow more than one student per academic year, but each student must be provided with one-on-one teaching and training. The learning objectives of the placement are made clear to the FMP teachers and students by the university. Further didactic courses and other training to improve teaching skills are encouraged and provided to the FMPs by the university but are not mandatory. Annual meetings for the FMPs are organized by the Institute of Primary Care to monitor progress/address any issues that may have arisen during the placements.

### Study Design and Recruitment

The design was a cross-sectional survey study, including all fourth-year undergraduate medical students at the University of Zurich who completed the mandatory placement in FMP during the period 2019–2022. Students chose the practices from those enrolled on a first-come, first-served basis. Basic information about the FMPs (name, address, and specialty) was available to students during online registration.

At the end of the placement, the students completed the mandatory online survey as part of the course requirement and as a formative evaluation by the university to monitor the quality of teaching in the FMPs. During the national Covid-19 lockdown, 18th March–26th April 2020, placement was suspended and evaluation was not mandatory.

### Data Description and Measurements

The survey, in German, comprised 40 items, grouped into 29 main questions, and investigated six themes: 1) organization of the placement, 2) course content, 3) teaching provided and the FMP learning environment, 4) patient–student interaction, 5) skills acquisition, and 6) course satisfaction. Within each section, key questions (screening questions) were defined as the main indicators for teaching placement quality to screen for FMPs that may need improvement. A copy of the original survey, translated into English, and a visual structure were provided (Supplementary Figures S1 and S2). Most of the items, 36, were defined on a Likert scale, from 1 (strongly disagree or not at all satisfied as applicable) to 6 (strongly agree or very satisfied); there were: one yes/no question about whether the travel time to the placement was reasonable, 3 open-ended questions about what the student liked most about the placement, benefited most from and suggested to improve, and comments on some questions related to themes 1) and 6).

Student information, like age, sex, nationality and grade was not collected.

The FMP specialty (pediatric or adult FMP) and urbanization of the FMP location (urban, suburban, or rural areas) were taken into account in the analysis. Urbanization was defined according to Eurostat’s degree of urbanization (DEGURBA), applied to Switzerland by the Swiss Federal Statistics Office.^28^

### Outcomes

The main outcome was the student evaluation score for each item within the 14 screening questions at the student level or averaged per FMP. The outcome subgroups were the FMP specialty and urbanization of the FMP location. Pairwise associations between items were examined. The second outcome was placement quality, defined as two groups of FMPs: benchmark and need to improve. These clusters were determined by the multidimensionality of the screening question scores and the number of students per FMP, as the latter could affect the capacity of the FMP and, therefore, the quality and reliability of the student evaluation. Finally, students’ perceptions were the third outcome, described through conceptual codes emerging from the qualitative analysis of the open-ended questions.

### Statistical Analysis

All analyses were carried out using the statistical package R version 4.1.0.^29^

Quantitative analysis of the Likert scale question items was performed: 1) at the student level, overall, stratified by FMP in the two quality groups and by year; 2) at the FMP level, overall and stratified by quality group, and by specialty and urbanization groups. FMPs in the two quality groups were identified using a k-means cluster analysis algorithm based on the spatial distance between the item average scores on the 14 screening questions and the number of students per FMP.

For each item, the results were defined as average scores when grouped by FMP or year. When performing group comparisons of these average scores, results were presented as median [interquartile range (IQR)] with p-values from the Wilcoxon-Mann–Whitney test or Kruskal–Wallis test, as appropriate, since the variable distributions were skewed. In case of missing data at the student-level item, available case analysis was performed, reporting the number of non-missing observations.

At the student level, Likert scale question scores were also categorized as disagree or not satisfied (scores 1–2); neutral (3–4); and agree or satisfied (5–6). Group comparisons were performed using chi-square or Fisher exact tests as appropriate.

The association between items was examined using pairwise Spearman rank correlations, which were reported between the average scores on the screening questions and on the question about the motivation to become a family medicine doctor and the number of students per FMP.

The total score (average by FMP), calculated as the sum of the scores of the screening questions, was also represented graphically and compared between the two quality groups.

Statistical significance was defined as p-value < 0.05.

### Qualitative Methods

Qualitative analysis of the open-ended questions and comments was performed using a framework analysis, which allows the inclusion of both sets in advance and emerging issues.^30^

Some of the quality criteria list,^31^ were identified in student answers and visualized through tag cloud figures, where more frequent tags appeared larger than the less frequent ones. Tags, assigned by the researcher from student texts, were used as conceptual codes to describe needs or suggestions for improvement, and the most liked things about the placement, barriers, or factors that negatively influenced the learning process and its evaluation. A specification of the main tags is provided (Supplementary Figure S3).

## Results

### Participation

During the academic years 2019–2022, a total of 713 students (145 in 2019–2020; 310 in 2020–2021 and 258 in 2021–2022) were included. The response rate was overall 81%. The students were allocated to 249 FMPs with median [IQR] of 2 [1,4] students per FMP. Only 20 (8%) were pediatric FMPs.

Most practices were located in the Zurich area (Supplementary Figure S4).

The majority of FMPs, 136 (55%), were located in the urban area and only 10 (4%) in rural areas. Almost all the students 709 (99.4%) reported that the travel time to the location was reasonable.

### Main Outcome: Students’ Evaluation

**Figure 1:**
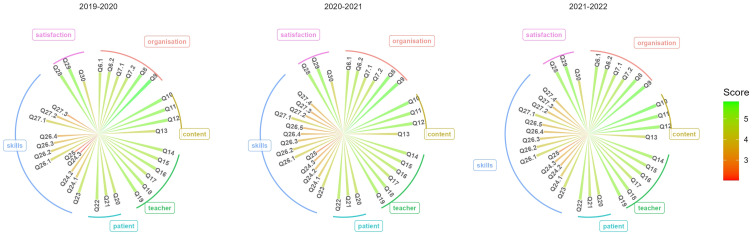
Student evaluation of placements in Swiss family medicine practice by year, period 2019–2022.

**Notes**: Average score was reported for each question. All questions were grouped in six categories evaluating the dimension of teaching quality: 1) placement organization; 2) course content; 3) teacher/family medicine practice learning environment; 4) patient-student interaction; 5) skills acquisition; 6) satisfaction. Each item was labelled as Q followed by the number in the original survey item list (see Supplementary Figure S1).

Almost all students were satisfied with the placement, 612 (86.2%), and even more with the teacher, 634 (89.3%). Almost half of them, 337 (47.5%), reported that the placement increased their motivation to choose FMP as a career (Table 1 and Supplementary Table S1). Almost all of the students, 680 (95.4%), agreed that the placement allowed them to obtain a realistic insight into the FMP work. Overall, 82% of the participants reported increased confidence after training for at least one clinical skill. The skill evaluation improved over time (Figure 1).

**Table 1:** Evaluations of Placements of Medical Students in Swiss Family Medicine Practice (2019–2022): Results of Screening Questions Overall and by Subgroups

		Student Level^a^	FMP Level	Benchmark^b^ Group	Need to Improve^b^ Group	p
		713	249	FMP=224 Students = 600	FMP=25 Students = 113	
1. Organization			Median [IQR]	Median [IQR]	Median [IQR]	
Communication with the teaching practice was easy	Q9	N (%)	6.00 [5.67, 6.00]	6.00 [5.97, 6.00]	5.25 [5.00, 5.50]	<0.001
Disagree		6 (0.8)		2 (0.3)	4 (3.5)	0.001
Neutral		21 (2.9)		15 (2.5)	6 (5.3)	
Agree		686 (96.2)		583 (97.2)	103 (91.2)	
2. Content						
The course gave me a realistic insight of the work in the FMP	Q10	N (%)	6.00 [5.50, 6.00]	6.00 [5.67, 6.00]	5.12 [5.00, 5.33]	<0.001
Disagree		5 (0.7)		0 (0.0)	5 (4.4)	<0.001
Neutral		28 (3.9)		7 (1.2)	21 (18.6)	
Agree		680 (95.4)		593 (98.8)	87 (77.0)	
I was introduced to the organization, set-up, workflows of a FMP	Q11	N (%)	5.75 [5.33, 6.00]	5.80 [5.50, 6.00]	4.83 [4.50, 5.00]	<0.001
Disagree		6 (0.8)		1 (0.2)	5 (4.4)	<0.001
Neutral		47 (6.6)		18 (3.0)	29 (25.7)	
Agree		660 (92.6)		581 (96.8)	79 (69.9)	
I experienced the most important core competencies^c^ of FMP	Q12	N (%)	5.80 [5.33, 6.00]	6.00 [5.50, 6.00]	4.75 [4.50, 5.00]	<0.001
Disagree		10 (1.4)		2 (0.3)	8 (7.1)	<0.001
Neutral		60 (8.4)		28 (4.7)	32 (28.3)	
Agree		643 (90.2)		570 (95.0)	73 (64.6)	
3. Teacher						
My teaching physicians showed me many examinations	Q14	N (%)	5.50 [5.00, 6.00]	5.50 [5.00, 6.00]	4.22 [4.00, 4.80]	<0.001
Disagree		31 (4.3)		6 (1.0)	25 (22.1)	<0.001
Neutral		99 (13.9)		69 (11.5)	30 (26.5)	
Agree		583 (81.8)		525 (87.5)	58 (51.3)	
My teaching physician checked and corrected my examination methods on the patient	Q15	N (%)	5.50 [5.00, 6.00]	5.50 [5.00, 6.00]	4.00 [3.00, 4.33]	<0.001
Disagree		56 (7.9)		22 (3.7)	34 (30.1)	<0.001
Neutral		107 (15.0)		74 (12.3)	33 (29.2)	
Agree		550 (77.1)		504 (84.0)	46 (40.7)	
My teaching physician gave me the opportunity to examine patients on my own	Q16	N (%)	5.50 [4.83, 6.00]	5.54 [5.00, 6.00]	4.11 [3.67, 4.80]	<0.001
Disagree		56 (7.9)		28 (4.7)	28 (24.8)	<0.001
Neutral		101 (14.2)		81 (13.5)	20 (17.7)	
Agree		556 (78.0)		491 (81.8)	65 (57.5)	
My teaching physician gave me regularly feedback	Q17	N (%)	5.00 [4.25, 5.60]	5.00 [5.00, 5.70]	3.75 [3.00, 4.00]	<0.001
Disagree		54 (7.6)		22 (3.7)	32 (28.3)	<0.001
Neutral		156 (21.9)		118 (19.7)	38 (33.6)	
Agree		503 (70.5)		460 (76.7)	43 (38.1)	
My teaching physician became a role model for me through his way of dealing with patients	Q18	N (%)	5.50 [5.00, 6.00]	5.67 [5.28, 6.00]	4.17 [4.00, 4.67]	<0.001
Disagree		25 (3.5)		9 (1.5)	16 (14.2)	<0.001
Neutral		86 (12.1)		47 (7.8)	39 (34.5)	
Agree		602 (84.4)		544 (90.7)	58 (51.3)	
I have learned a lot in dealing with patients through my teaching physician	Q19	N (%)	5.67 [5.00, 6.00]	5.75 [5.31, 6.00]	4.00 [4.00, 4.50]	<0.001
Disagree		20 (2.8)		7 (1.2)	13 (11.5)	<0.001
Neutral		91 (12.8)		48 (8.0)	43 (38.1)	
Agree		602 (84.4)		545 (90.8)	57 (50.4)	
4. Patient						
I was made aware of social problems in the context of the patient visit	Q22	N (%)	5.22 [5.00, 6.00]	5.33 [5.00, 6.00]	4.38 [4.00, 4.67]	<0.001
		N=712		N=600	N=112	<0.001
Disagree		17 (2.4)		6 (1.0)	11 (9.8)	
Neutral		131 (18.4)		97 (16.2)	34 (30.4)	
Agree		564 (79.2)		497 (82.8)	67 (59.8)	
5. Skills						
I could practice how to take a medical history	Q23	N (%)	5.00 [4.00, 5.60]	5.00 [4.00, 5.69]	4.00 [3.00, 4.40]	<0.001
		N=711		N=599	N=112	<0.001
Disagree		102 (14.3)		70 (11.7)	32 (28.6)	
Neutral		167 (23.5)		135 (22.5)	32 (28.6)	
Agree		442 (62.2)		394 (65.8)	48 (42.9)	
6. Satisfaction						
How satisfied are you with the course in the teaching practice?	Q28	N (%)	5.83 [5.25, 6.00]	6.00 [5.50, 6.00]	4.25 [4.00, 4.50]	<0.001
		N=710		N=598	N=112	<0.001
Not satisfied		20 (2.8)		6 (1.0)	14 (12.5)	
Neutral		78 (11.0)		34 (5.7)	44 (39.3)	
Satisfied		612 (86.2)		558 (93.3)	54 (48.2)	
How satisfied are you with your teaching physician?	Q29	N (%)	6.00 [5.50, 6.00]	6.00 [5.67, 6.00]	4.33 [4.00, 4.56]	<0.001
		N=710		N=598	N=112	<0.001
Not satisfied		22 (3.1)		7 (1.2)	15 (13.4)	
Neutral		54 (7.6)		19 (3.2)	35 (31.2)	
Satisfied		634 (89.3)		572 (95.7)	62 (55.4)	
Number of student followed (2019–2022)			2.00 [1.00, 4.00]	2.00 [1.00, 3.00]	3.00 [1.00, 6.00]	0.089

**Notes**: ^a^At the student level, scores were categorized as disagree or not satisfied (Likert scale 1–2); neutral (Likert scale 3–4); agree or satisfied (Likert scale 5–6) and reported as number(percentage), N(%), with p-value (p) from the chi-square test. Median and interquartile range [IQR] of item average scores, in scale 1–6, at the family medicine practice level, were reported with p-values from the Wilcoxon-Mann–Whitney test. ^b^Benchmark and need to improve groups were identified through the k-means algorithm based on the screening questions and the number of students followed. ^c^Prevention and counselling, acute treatments, chronic illness care, guide function/coordination with the healthcare system.

**Abbreviation**: FMP, family medicine practice.

The skills acquisition questions reported the lowest percentages of agreement even when taking into account the differences between pediatric and adult FMP (Table 1 and Supplementary Table S1). For example, 299 (42.1%) students in all FMPs agreed to have been able to carry out vaccination, 350 (54.3%) students located in the adult FMPs agreed to have been able to perform blood draw, and 170 (26.4%) to acquire other skills, that is, applying and changing bandages. In addition to the differences in the skills provided and specific course content (Supplementary Tables S2 and S3), a higher average score, relative to the examinations shown in advance by the doctor, was observed in pediatric FMPs compared to adult FMPs, 5.90 [5.50, 6.00] vs 5.50 [5.00, 6.00], p=0.023.

FMPs in urban areas appeared worse than FMPs in rural and suburban areas regarding the following aspects: giving insight into FMP work, organization/management, core competences, giving a professional role model, communication with patient, some additional skills like age-appropriate check-up, raising the neurological status, X-ray analysis, student satisfaction, and motivation to work in FMP (Supplementary Tables S2 and S3).

**Figure 2:**
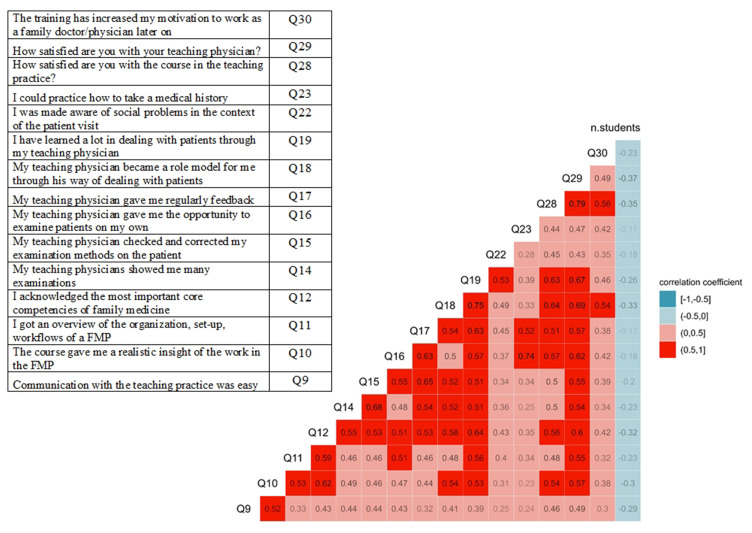
Correlation matrix (pairwise Spearman rank correlation coefficients) between item average scores from student evaluation of Swiss family medicine practice placements, period 2019–2022.

**Notes**: The Figure showed how the screening question scores, the question about the motivation to become a family medicine doctor (Q30) and the number of student per family medicine practice (FMP) were correlated. Item scores were averaged at FMP level. In the legend, correlation greater than 0.5, from moderate to strong, was highlighted in red. Negative correlation, not greater than −0.5, from moderate to strong, was highlighted in blue.

The highest positive correlation (0.79) was found between satisfaction with the course and with the teacher (Figure 2). Both aspects of satisfaction positively correlated with the professional role model, 0.64 and 0.69, the conveyed way of dealing with patients, 0.63 and 0.67, insight into the FMP work, experience with core competences, student independence during patient examinations and regular feedback by supervisors. The number of students per FMP had a negative low correlation with both aspects of satisfaction, −0.35 and −0.37. Motivation to pursue a career in FMP was positively correlated with satisfaction with the course (0.56) and the professional role model (0.54).

### Second Outcome: Benchmark vs Need to Improve FMPs

A cluster of 25 (10%) FMPs that need to improve, different from the benchmark group, in terms of performance, was identified (Supplementary Figure S5). In this cluster, only one (4%) FMP was pediatric; one (4%) was in rural areas and six (24%) in suburban areas. The need to improve group, compared to the benchmark, had significantly lower score in each screening question and in all other items, except electrocardiogram skills, and in the total score (Table 1, Supplementary Table S1 and Figure S6).

### Third Outcome: Students’ Perceptions

**Figure 3:**
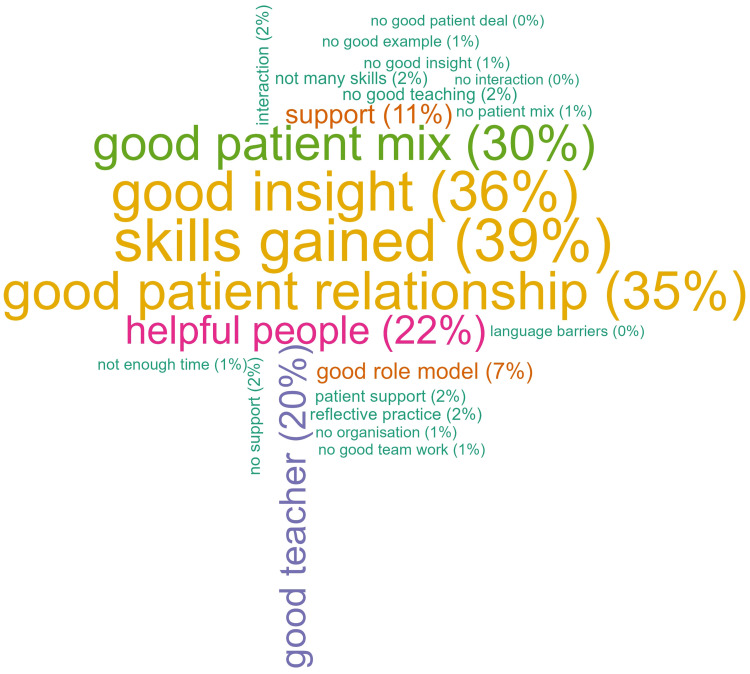
Tag cloud of what medical students most liked about the Swiss family medicine placement (2019–2022).

**Notes**: Tags assigned from the answers to the questions Q31: “What did you like most about the training?” and Q32: “What did you benefit the most from placement?” were combined together to catch the general student perception about the placement. For each tag, the percentage of all 713 students, was reported, though 575 students answered the open-ended questions.

The main themes, with students’ quotes arising from open-ended questions, were reported (Table 2). In particular, language barriers and a stressed or struggling team were examples of factors that negatively influenced the learning environment. By contrast, an enthusiastic or motivated team was inspiring. However, sharing experiences with other students influenced individual evaluations. The tags assigned from the themes highlighted that students most liked the skills gained (39%), the good insight into FMP (36%), and the good relationship with the patients (35%) (Figure 3). A minority of the students (19%) did not answer all open questions. The majority of the students, 432 (61%) did not provide suggestions on what could be improved in the placement.

**Table 2:** Evaluations of Placements of Medical Students in Swiss Family Medicine Practice (2019–2022): Qualitative Analysis

Themes	Quotes	Tags
**Needs**		
More skills/practice	I would have been happy if the course with my teaching physician had been more practically oriented. With a few exceptions, I could actually only sit in the corner and listen and thus not develop my practical skills further.	Skills/practice
More independence though feedback	…Unfortunately, I could only perform very few examinations independently, but I think that this is justified due to pediatric and adolescent medicine.	Autonomy/feedback
More time, questions or explanations	…However, I found there was little time overall to ask questions or get comprehensive explanations, which was a bit of a shame.	Time/commitment
Definitions of goals / guidelines	The course is highly dependent on the wishes and ideas of the teaching doctor. If I had been less lucky with my teaching doctor, the course could have been much more monotonous. Perhaps stricter guidelines and manuals would be useful.	Objectives
**Most Like**		
Good insight / welcome into the family medicine team.	I got a good insight into the day-to-day work in the practice.	Good insight
	… I personally really liked it. The doctors, the team and the organization were all very nice and communicative and also willing to show you a lot. Just the range from the many specialties was nice and showed that they were interested in giving me a big overview…	Good insight, helpful people
Dealing with patients / establishing a good relationship.	I liked seeing the whole spectrum of patients from young to old and from almost healthy to seriously ill. I am impressed by the relationship the family doctor has built with his patients and how insanely quickly he knows the diagnosis. Every patient comes in with something completely different and being able to narrow down this “anything is possible” so quickly, while still incredibly difficult, seems to me now much more feasible than before this course.	Good patient relationship, good patient mix
Teacher enthusiasm / commitment	There were three doctors in the practice team who were all very enthusiastic about showing me the most exciting cases and always explained everything to me in a very motivated manner. The assistants were also extremely open-minded, friendly, upbeat and motivating. In every internship day the atmosphere was very motivating and it was very educational!	Good teacher, good insight, helpful people
Skills acquired	Drawing blood, practicing infusions and injections with the MPAs: it was the first time in my studies where I could do something like that and it was great how the team took time for me. I also benefited greatly from being able to conduct anamneses and clinical examinations independently with patients and document my findings directly.	Skills gained
**Difficulties/barriers**		
Influencing patient relationship	…In my case, it was unfortunately the case that 80% of the patients spoke Portuguese (the teaching physician as well) and I do not understand this language. Thus, the patient contact was severely limited.	Language barriers
	…However, the team was stressed. I also heard several complaints about the family doctor’s way of working and time management. I found this stress, which the patients also noticed, to be quite negative.	No good insight, no good example, no good team work
Influencing learning process	Unfortunately, there were many open conflicts between the teaching physician and her assistants. In many cases, I felt involved in these conflicts against my will. Although I could very well understand the teaching physician’s point of view, these circumstances had a negative effect on my learning success.	No good insight, no good example, no good team work
**Other**		
Sharing experience influencing evaluation	I actually liked it a lot. Everyone was very nice. However, I find that I was allowed to conduct fewer anamneses (approx. 3x) and examinations (4–5x) compared to colleagues students.	Skills/practice
Placements increasing student motivation	Before the placement I was not so enthusiastic about family medicine, now I am	Good insight
	I really liked the one-to-one support on this course. I also really liked the practice in general: all the staff were incredibly helpful and open. You were always allowed to ask questions and even tell them when you reached your limits. I was allowed to do a lot of hands-on work and also examine myself, do an ECG, take an ultrasound in my hand and take anamnesis. Thanks to the good supervision, I never felt insecure or overwhelmed. All in all, I really enjoyed the course and was able to take a lot with me for my future.	Good teacher, good insight, helpful people, skills gained, support

**Notes**: Main themes, with quotes, arising from students’ comments and the open-ended question Q31: What did you like most about the training? Q32: What did you benefit the most from placement? Q33: What can be improved in the course?

The three main needs were more independence and feedback when practicing skills in supervision (10%), more possibility of practicing skills rather than observing supervisors doing them (7%), and better identification and sharing of the learning objectives with the FMP (5%) (Supplementary Figure S7).

## Discussion

### Main Findings

The main findings of the study are as follows: 1) almost all students were satisfied with the placement, and even more with the teacher; 2) almost all students agreed that the placement allowed them to get a realistic insight into the FMP work; 3) a cluster of 10% of FMPs should improve the FMP learning environment; 4) half of the students reported having practiced the clinical skills, and some of them would like to practice more; 5) students liked to establish patient relationships; 6) the motivation to pursue the FMP career was positively correlated with the satisfaction with the course and the conveyed professional role model.

### Comparisons with Existing Literature

Our findings confirm previous findings that placements in FMP offer good learning opportunities for medical students and provide a realistic insight into FMP with high student satisfaction.^3^,^4^,^6^,^7^,^19–23^,^25^

Some researchers have determined that giving feedback, teaching the trainee to carry responsibility, being able to communicate, being interested in the trainee, and being able to detect the individual learning needs are some characteristics of a good trainer in general practice.^32^ Moreover, the ability to convey knowledge and to show how things should be done are indicators of good clinical teaching and supervision.^33^

All these skills were evaluated in our survey, specifically for FMP placement and its objectives, within the screening questions to identify FMP clusters that need to improve the FMP learning environment. As the quality of teaching also depends on the time and effort dedicated to each student to provide feedback and explanations,^19^ our benchmark also considered the number of students followed by the same practice. However, difficulties during the placement due to many different reasons could affect the quality of teaching provided.^6^,^19^,^34^,^35^ In the current study, participants acknowledged conflicts within the practice team, stressful environments, and language barriers that interfered with learning and training. Although our cluster analysis identified 10% of FMPs that should improve the FMP learning environment, only a negligible proportion of students reported, in open-ended questions, that they did not have a good teacher using the criteria list.^31^ This is consistent with other studies.^4^

According to our findings, there is evidence^6^,^24^ that students in FMP enjoy opportunities to conduct their own consultations and develop additional skills, and they value receiving direct feedback, particularly on physical examination skills. In our study, as in a study from Germany,^22^ sometimes students felt like passive observers during placement, although the skill evaluation appeared to improve over time. Moreover, in line with other studies,^20^ clinical skills practicing in rural placement FMPs were at least as good as, and often better than, those of students in urban settings. This might be due to greater involvement with older patients or the provision of more acute care in rural FMPs compared to FMPs in other areas.

Students valued the opportunity to see a range of different patients and to learn how to improve communication with patients, particularly by observing how supervisors might handle difficult consultations, ie giving bad news or asking sensitive questions. Students also gained insight into the social context of healthcare by understanding patients’ family circumstances. This is consistent with previous research.^4^,^7^

Previous studies have shown that early exposure to FMP has a positive impact on student attitudes towards family medicine^24^,^36^ and the conveyed FMP role model appears to be of greater importance in influencing the career choice in FMP.^37^ In our study, nearly half of the students reported that the placement increased their motivation to choose FMP as a career. Moreover, motivation to choose a career in FMP mostly correlated with the student satisfaction with the placement and the conveyed professional role model.

### Strengths and Limitations

Although studies have investigated medical students’ perspectives and needs in FMP,^3^,^4^,^6^,^7^,^19–25^ to the best of our knowledge this is the first study in Switzerland. The novelty of this study is the application of a cluster analysis algorithm (k-means) to find a benchmark placement quality group.

A strength of this study is the high response rate (81%). However, the main limitation is that our survey was not validated, and with a cross-sectional design, we could not investigate causality. Teaching placement quality was defined using indicators based on the undergraduate students’ first impressions of the FMP setting. We are aware that students might not be able to judge what a realistic insight into FMP is, since they lack comparison and experience and might need more time to evaluate it. However, students’ perceptions of their learning experience are associated with academic achievement^38^ and are a valid measure of teaching quality.^39^ Analogously, the results about skills and competencies were based on self-assessed measurements, but we did not have information about student characteristics or academic performance that could be associated with the evaluation. We also did not consider other FMP characteristics, such as single/double practice or experience of teaching doctors in practice. In addition, practicing clinical skills was not considered within the benchmark quality, nor properly assessed as a learning objective, as it cannot be standardized in FMP placements due to the diversity of teaching practices.

When comparing our study with the international context,^26^ we should also consider that the duration and organization of placements in our study are different from other settings, which might limit the generalization of our findings. Lastly, not having information about students’ expectations prior to the placement was also a limitation, as we could not determine their effect on the students’ satisfaction with the placement and with the motivation for the FMP career.

### Implication for Practice and Research

The study is relevant to the medical education provision and then to the FMP because it described students’ experiences in FMP and used a methodology to evaluate the quality of placements. To motivate more students to actively pursue a career in FMP, it is essential that undergraduate family medicine teaching and training is carefully planned to emphasize the positive aspects of FMP. Given that medical students consider part-time work, autonomy and relationships with patients as important career determinants, helping students understand how these determinants relate to family medicine may increase their interest in the profession.^17^ Therefore, the placement is an opportunity to promote the FMP career and may also improve the quality of healthcare provided.^2^,^4^,^26^ Since in our study the motivation to choose a career in FMP mostly correlated with the students’ satisfaction with the placement and the conveyed professional role model, regular evaluation of FMP placements through students’ views is very important to positively influence students’ attitudes towards FMP. On the other hand, to identify FMPs that could benefit the most from training or programs to improve their learning environment makes the improvement process more efficient. In fact, universities could focus on participating FMPs that need to improve the placement by asking them to attend specific courses or to meet specific criteria to continue offering student placements (eg from our findings, not more than two students per academic year, offering a different patient mix and a broad treatment range, giving students regular feedback). This could also improve the future recruitment of FMPs, who should meet these specific criteria to participate that, according to our findings, are associated with better learning environments. The methodology used in this study to identify these FMPs is intended for evaluation within a quality–improvement process, and not to discriminate between “good” and “bad” placements. Moreover, guidelines and standard definitions of the learning objectives of teaching placements, nationally and internationally, would improve the quality of placements and comparability among countries,^26^,^40^ but the process of selecting practices and the communication of educational objectives and skills requirements between the university, practices, and students should not be neglected. On the other hand, teaching quality depends on individual needs and expectations,^33^ which are also culture-specific.^39^ The main message from this study, based on qualitative feedback from students, is to improve placements so that students gain a wide range of clinical skills, are involved in patient consultations, have more responsibility where possible and receive appropriate feedback. Further research is needed to better investigate all factors that might influence student perception and evaluation together with the motivation to choose FMP as a career. In fact, some students reported in the open-ended comments that the placement and the teacher did not interfere with their motivation because from the beginning they were not interested in the profession.

## Conclusion

Evaluating placements in FMP at the undergraduate level is essential for adapting medical education to the evolving needs of the healthcare system, particularly to address the shortage of family physicians. This study described students’ experiences in FMP and used a methodology to properly identify FMPs that could benefit the most from training or programs to improve their learning environment. Our study suggests that expanding the teaching of clinical skills, involving students in patient consultations, giving them more responsibility where appropriate, and providing them with regular feedback would have a positive impact on students’ experiences and could increase their interest in working in FMP.

## Data Availability

The datasets used and/or analyzed during the current study are available from the corresponding author upon reasonable request.
